# Nociceptive tolerance is improved by bradykinin receptor B1 antagonism and joint morphology is protected by both endothelin type A and bradykinin receptor B1 antagonism in a surgical model of osteoarthritis

**DOI:** 10.1186/ar3338

**Published:** 2011-05-16

**Authors:** Gabriel N Kaufman, Charlotte Zaouter, Barthélémy Valteau, Pierre Sirois, Florina Moldovan

**Affiliations:** 1 Orthopaedic Molecular Biology Laboratory, Sainte-Justine Hospital Research Centre, 3175 Côte Sainte-Catherine, Montreal, QC, H3T 1C5, Canada; 2 Paediatric Mechanobiology Laboratory, Sainte-Justine Hospital Research Centre, 3175 Côte Sainte-Catherine, Montreal, QC, H3T 1C5, Canada; 3 IPS Thérapeutique, 3201 Jean-Mignault, Sherbrooke, QC, J1E 4K8, Canada; 4 Faculty of Dentistry, Université de Montréal, PO Box 6128 Stn CV, Montreal, QC, H3C 3J7, Canada

## Abstract

**Introduction:**

Endothelin-1, a vasoconstrictor peptide, influences cartilage metabolism mainly via endothelin receptor type A (ETA). Along with the inflammatory nonapeptide vasodilator bradykinin (BK), which acts via bradykinin receptor B1 (BKB1) in chronic inflammatory conditions, these vasoactive factors potentiate joint pain and inflammation. We describe a preclinical study of the efficacy of treatment of surgically induced osteoarthritis with ETA and/or BKB1 specific peptide antagonists. We hypothesize that antagonism of both receptors will diminish osteoarthritis progress and articular nociception in a synergistic manner.

**Methods:**

Osteoarthritis was surgically induced in male rats by transection of the right anterior cruciate ligament. Animals were subsequently treated with weekly intra-articular injections of specific peptide antagonists of ETA and/or BKB1. Hind limb nociception was measured by static weight bearing biweekly for two months post-operatively. Post-mortem, right knee joints were analyzed radiologically by X-ray and magnetic resonance, and histologically by the OARSI histopathology assessment system.

**Results:**

Single local BKB1 antagonist treatment diminished overall hind limb nociception, and accelerated post-operative recovery after disease induction. Both ETA and/or BKB1 antagonist treatments protected joint radiomorphology and histomorphology. Dual ETA/BKB1 antagonism was slightly more protective, as measured by radiology and histology.

**Conclusions:**

BKB1 antagonism improves nociceptive tolerance, and both ETA and/or BKB1 antagonism prevents joint cartilage degradation in a surgical model of osteoarthritis. Therefore, they represent a novel therapeutic strategy: specific receptor antagonism may prove beneficial in disease management.

## Introduction

Osteoarthritis (OA) is characterized by a progressive destruction of articular cartilage accompanied by subchondral bone remodeling, osteophyte formation, and synovial membrane inflammation [1]. Clinically, this disease progresses slowly and principally affects the hands and large weight-bearing joints. Pain is the primary complaint of patients with OA. Its etiology is multifactorial: subchondral bone can have micro-fractures, osteophytes can cause stretching of peri-osteal nerve endings, ligaments may be stretched, the joint capsule can be inflamed or distended, the synovium may be inflamed, and muscles may spasm [2]. Furthermore, neo-innervation of joint tissue concurrent with angiogenesis [3,4] may contribute to deep joint pain. Further understanding of the molecular mechanisms behind these effects should provide avenues towards targeted disease-modifying or -slowing treatments [5,6].

We have previously shown that endothelin-1 (ET-1), a 21-amino-acid potent vasoconstrictor peptide, plays a major role in OA pathogenesis. It reduces cartilage anabolism by inhibiting collagen and proteoglycan synthesis [7]. It causes matrix metalloproteinases one and thirteen to be synthesized and activated in OA cartilage [8]. ET-1 also causes excessive production of nitric oxide, which is generated as the result of an increase in inducible nitric oxide synthase levels [9]. These effects occur mainly via endothelin receptor type A (ETA) [10]: it is expressed in articular tissue by chondrocytes, synoviocytes, and endothelial cells, where it plays a significant role in cartilage and bone metabolism [11,12]; ETA also potentiates inflammatory joint pain induced by ET-1 [13,14].

ET-1 affects vascular homeostasis via the renin-angiotensin-aldosterone system [15]. Through cross-talk with the kallikrein-kinin system [16], it can also mediate kinin-induced pain and inflammation. Bradykinin (BK), the inflammatory nonapeptide vasodilator, has also been implicated in OA pain and inflammation. It is generated in OA synovium, as in all inflamed tissue; it also is released due to the increased vascular pressure in subchondral bone [17]. BK binds two receptors, bradykinin receptor B1 (BKB1) and bradykinin receptor B2 (BKB2). The effects of BK in OA occur largely via BKB1, a receptor implicated in articular nociception [18,19] and pro-inflammatory reactions [20]. BKB1 also potentiates the effects of other pro-inflammatory mediators such as cytokines and prostaglandins. BKB2, though it has been implicated in nociceptor sensitization in OA [17,19], may be less relevant as a therapeutic target in the context of a chronic inflammatory response. It is constitutively expressed to a large extent, and is primarily involved in the acute phase of inflammation [21,22]. In contrast, BKB1 is up-regulated in chronic inflammatory conditions, its expression often induced secondary to inflammatory mediator release [22-24].

Antagonism of ETA and/or BKB1 may represent a novel therapeutic option to alleviate, and perhaps prevent or reverse, the pain, inflammation, and tissue damage that occur as OA progresses from an acute to a chronic state. We hypothesize that ETA and BKB1 antagonism will diminish OA progress in a synergistic manner. In the present work, we describe a preclinical study of the efficacy of treatment of surgically induced OA with ETA and/or BKB1 peptide antagonists, using an established rat model of the disease. We found that BKB1 antagonist treatment diminished hind limb nociception, and both ETA and/or BKB1 antagonism protected joint radiomorphology and histomorphology. This demonstrates that ETA and BKB1 receptor expression is involved in OA pathogenesis, and that specific receptor antagonism may prove beneficial in OA disease management.

## Materials and methods

### Rat model of osteoarthritis

#### Animals

Eight-week-old male Lewis rats were purchased from Charles River Canada (Saint-Constant, Quebec) and housed under standard conditions. All procedures were approved by the Sainte-Justine Hospital Research Centre animal ethics committee and conformed to Canadian Council on Animal Care guidelines [25].

#### Study design

The study was conducted as a fractional factorial experiment. Animals were randomly assigned to one of three surgery conditions: anterior cruciate ligament transection (ACLT), sham surgery, or no surgery (negative control). Subsequently, animals were assigned to one of four treatment groups, as detailed below (Table 1). Sample size was *n *= 6 per group.

**Table 1:** Experimental groups

Group number	Surgery	Treatment
1	None	Saline
2	Sham	Saline
3	ACLT	Saline
4	ACLT	BQ-123
5	ACLT	R-954
6	ACLT	BQ-123+R-954

Six experimental groups were designated in the fractional factorial study, with six subjects per group.

#### Surgical technique

OA was induced by surgical transection of the right anterior cruciate ligament. The procedure was modified from previously published reports [26-29], and is described in detail in Additional file 1. Briefly, animals were anaesthetized and subjected to either anterior cruciate ligament transection or sham surgery. One group of animals, kept as negative controls, were not operated upon.

### Drug treatment

Over the course of two months post-operatively, animals were treated by weekly intra-articular injections of ETA and/or BKB1 specific peptide antagonists: BQ-123 (ETA antagonist; Sigma-Aldrich, Oakville, Ontario) [30,31], R-954 (BKB1 antagonist; a kind gift from Pierre Sirois, IPS Thérapeutique, Sherbrooke, Quebec) [32,33], both, or saline vehicle, was injected into the right knee at a dose of 30 nmol in a volume of 50 *μ*L. Injections were performed under isoflurane anaesthesia, using a 28G needle; the procedure is described in detail in Additional file 1. Chemical structures of the antagonists are depicted in Additional file 2. Doses were based upon previously published reports [14,19].

### Static weight bearing

Over the course of the study, animal nociception was evaluated biweekly by the static weight bearing test. A static weight bearing apparatus was reverse-engineered from previously published reports [34-36], designed, and machined by Usinage FB (Le Gardeur, Quebec). Design diagrams and photos are appended in Additional files 3 and 4.

After conditioning, animals were introduced to the apparatus and restrained in a plexiglass chamber with an angled base, such that each hind paw rested on a separate force plate connected to a load cell. The weight in grams distributed on each hind limb was recorded by a computer software interface (Futek USB software interface version 2.10). The static weight bearing distribution of each animal was recorded for 30 seconds; each data point was then taken as the mean of three 30-second readings. Data were transferred off-line to a personal computer, and the weight bearing on the right hind limb as a percentage of total weight bearing on both hind limbs was calculated by the following equation [37]:

All values are given as mean ± standard deviation (SD) per experimental group.

#### Statistics

Static weight bearing data were analyzed by repeated measures analysis of variance (ANOVA), which compares the global differences between groups of response profiles measured on the same subjects repeatedly over the course of the study [38,39]. Test values were taken as the dependent variable and treatment group as the independent variable, with the animal as the grouping factor. Sphericity was confirmed with Mauchly's *W *test. Tukey multiple comparisons testing was used to establish significance in between groups, with directionality taken from the sign of the mean difference. *P*-values less than 0.05 were considered statistically significant. Analyses were conducted using R (version 2.12.1) [40].

### Euthanasia and sample preparation

At four or eight weeks post-surgery, animals were sacrificed by cardiac puncture under deep isoflurane anaesthesia. The right knee was dissected, and 40-mm-long samples were cut and stored in phosphate-buffered saline until imaged by digital micro-X-ray (DX) and/or micro-magnetic resonance (MR). Samples were dissected the same day as the radiological scans.

### Digital micro-X-ray

All knee samples were X-rayed using a Faxitron MX-20 specimen X-ray system (Faxitron X-Ray Corporation, Lincolnshire, IL). Anteroposterior and lateral views were acquired at 5 × magnification (10 × 10 *μ*m pixel size) using a dose of 26 kV for 6 seconds. Images were analyzed using OsiriX software (version 3.7.1) [41]. Radiological evidence of joint degradation was scored by two blinded examiners using an OA radiological score modified from Clark *et al. *[42] and Esser *et al. *[43]. Bone demineralization, subchondral bone erosion, and heterotopic ossification were all scored on a scale from zero (normal) to three (marked degenerative changes). Total scores were calculated by summing the individual scores for each index, with a maximum possible score of nine.

#### Statistics

OA radiological scores were statistically analyzed by one-way ANOVA, with total scores taken as the dependent variable and treatment group as the independent variable. Pairwise post-hoc testing with Holm correction was used to establish significance in between treatment groups. *P*-values less than 0.05 were considered statistically significant. Analyses were conducted using R (version 2.12.1) [40].

### Micro-magnetic resonance imaging

#### Image acquisition

A subset of animals were sacrificed four weeks post-operatively and their right knees were imaged by micro-MR. Imaging was performed using a Bruker PharmaScan (Ettlingen, Germany) 7 Tesla MR scanner at the McGill University Small Animal Imaging Lab (Montreal, Quebec). Knee samples were placed in a custom-made support inside a 15-mL centrifuge tube, which was then filled with the MR-inert buffer FC-770 (3M Fluorinert Electronic Liquid). Samples were introduced into a ^1^H mouse brain radio frequency (RF) coil (inner diameter 22 mm), and centered in the magnet. The RF coil was tuned and matched to the sample, and the magnet was then shimmed. The system was controlled via Bruker ParaVision software (version 5.0).

Positioning was confirmed with a tri-pilot rapid scan, which was then used to place 14 coronal slices for two-dimensional anatomical scanning of the joint using a rapid acquisition with relaxation enhancement (RARE) multiecho spin echo pulse sequence (TurboRARE). Scan parameters were as follows: repetition time (TR) = 3500 milliseconds (ms), echo time (TE) = 36 ms, echo train length (ETL) = 8, slice thickness = 500 *μ*m, acquisition matrix = 384 × 384, and number of averages = 4. Voxel size was . These scans were then repeated in the sagittal projection.

Once these scans were acquired, one 1-mm-thick axial slice was placed in the center of the knee joint in order to scan the articular cartilage with a series of multislice multiecho (MSME) T_2_-weighted pulse sequences. Scan parameters were TR = 3500 ms, ETL = 1, acquisition matrix = 192 × 256, with voxel size of 156.25 × 156.25 × 1000 *μ*m. 16 different TE were used: 10, 20, 30, 40, 50, 60, 70, 80, 90, 100, 110, 120, 130, 140, 150, and 160 ms.

Total scan time was roughly 1 hour per sample. Scan sequences were based on previously published reports [44].

#### Image processing and analysis

After acquisition, images were analyzed using OsiriX software (version 3.7.1) [41]. Anatomical TurboRARE images were examined for correct depiction of anatomical features of the knee joint, and to confirm ACLT where applicable. As well, images were analyzed for signs of cartilage decay, indicated by lower signal intensity of the articular surfaces. The MSME-T_2 _images were aligned into an image stack, and regions of interest, corresponding to the articular cartilage, were manually drawn and propagated throughout the stack. A mean T_2 _fit map was then automatically generated by fitting the signal intensity to the spin-spin relaxation signal decay equation:

where signal intensity *S *is defined as a function of echo time TE, and is related to the spin density *M*_0 _and the transverse relaxation time T_2_. The equation was solved for the mean T_2 _value over the 16-image stack by using least-squares single-exponential curve-fitting, with initial guesses of *M*_0 _= signal intensity at 10 ms and T_2 _= 30 ms, in order to guarantee rapid convergence [44]. OsiriX then generated a T_2 _fit map graph with regression line and values for T_2 _and *M*_0_.

### Histology

After radiological examination, knee samples were fixed in 10% neutral buffered formalin for two weeks, decalcified with RDO Rapid Decalcifier (Apex Engineering Products, Aurora, Illinois) for three days, circulated, and embedded in paraffin. Five-micron sagittal sections were acquired from the middle of the knee joint. Histomorphological staining was performed as previously described [45]: slides were deparaffinized, rehydrated, stained with Safranin O (which colors proteoglycans red), counterstained with Fast Green FCF (which colors proteins green) and with Weigert's hematoxylin (which colors nuclei black), dehydrated, cleared, and mounted in Permount. Representative digital photomicrographs were acquired with a Leica DM R microscope (Wetzlar, Germany) fitted with a QImaging Retiga 1300 B camera (Surrey, British Columbia), controlled by QCapture software (version 2.95.0). Images were captured at 50 × (low-power) or 200 × (high-power) magnification, and subsequently color-matched and balanced using Adobe Photoshop CS3.

### Histopathological scoring

Four slides from each condition were scored by two blinded examiners using the Osteoarthritis Research Society International (OARSI) histopathology assessment system [46], which assigns numeric values to grade, or depth progression into cartilage (0-6), and stage, or extent of joint involvement (0-4); multiplying grade and stage yields a total OA score with a maximum value of 24. Scores were averaged in between the two examiners; inter-examiner variation was within ± 5%.

#### Statistics

OARSI scores were statistically analyzed by one-way ANOVA, with total scores taken as the dependent variable and treatment group as the independent variable. Pairwise post-hoc testing with Holm correction was used to establish significance in between treatment groups. *P*-values less than 0.05 were considered statistically significant. Analyses were conducted using R (version 2.12.1) [40].

### Immunohistochemistry

Additional 5-micron sections were processed for immunohistochemical detection of type II collagen. Slides were deparaffinized, rehydrated, and washed in phosphate-buffered saline (PBS). Sections were incubated in 2 mg/mL hyaluronidase for 30 minutes at 37°C, followed by permeabilization with 0.3% Triton X-100 for 30 minutes at room temperature. Endogenous peroxidase activity was then quenched with 2% hydrogen peroxide in PBS for 15 minutes. Sections were blocked with normal mouse serum (Vector Laboratories, Burlingame, California) for 1 hour, after which they were blotted and then incubated with monoclonal mouse anti-rat type II collagen (clone SPM239; Spring Bioscience, Pleasanton, California) for 18 hours at 4°C. Sections were then washed in PBS, incubated with biotinylated anti-mouse IgG (Vector) for 1 hour at room temperature, and stained using the avidin-biotin complex method (Vectastain ABC kit; Vector). Color was developed using 3,3'-diaminobenzidine (Dako Diagnostics, Mississauga, Ontario) containing hydrogen peroxide. Slides were counterstained with Harris modified hematoxylin, dehydrated, cleared, mounted, and examined by light microscopy as described above.

## Results

### ETA and BKB1 antagonism ameliorates OA nociceptive tolerance

**Figure 1:**
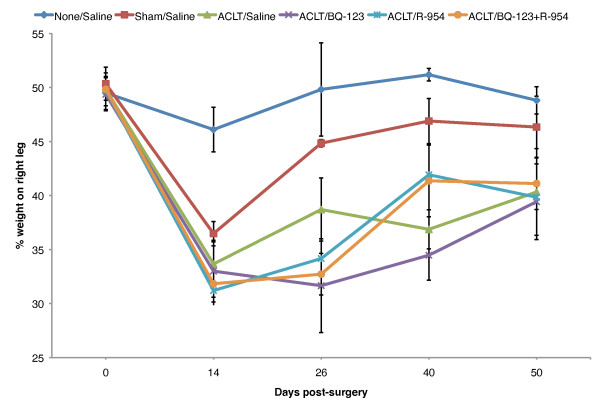
**ETA and/or BKB1 antagonist treatment improves static weight bearing tolerance**. Static weight bearing tolerance was measured repeatedly at defined time points over the course of the study. Data are presented as mean ± SD per experimental group (*n *= 6), of weight on the right leg as a percentage of total weight on both hind limbs. Day 0, baseline pre-operative values. Repeated measures analysis of variance with Tukey post-hoc (Table 2) indicated that BKB1 antagonist treatment significantly ameliorated nociceptive tolerance in ACLT animals over the study period, as compared to saline-treated positive controls.

To determine the effects of ETA and/or BKB1 local antagonist treatment on nociception in a surgical OA model, the static weight bearing asymmetry of the animals was measured repeatedly over the course of the study (Figure 1). Pre-operative baseline values for all groups indicated hind limb weight bearing symmetry (49.89 ± 0.42%). Unoperated vehicle-treated animals showed no important changes in hind limb weight bearing from baseline pre-operative values over the course of the study, staying roughly within ±4% of even weight distribution. Sham-operated vehicle-treated animals displayed an initial weight bearing imbalance 14 days post-operatively (36.47 ± 1.12%), but recovered weight bearing symmetry quickly thereafter (44.84 ± 0.33% by day 26 post-operatively). ACLT saline-treated animals showed significant weight bearing imbalance two weeks post-operatively, down to 33.66 ± 2.05% weight on the right leg, suggesting severe nociception. All animals had similar nociceptive tolerance at the last measured time-point (day 50 post-operatively), indicating nociceptive adaptation, but drug-treated animals were able to recover faster than saline-treated animals (up to 40.54 ± 3.36% weight on right leg by day 40 post-operatively, for BQ-123 and R-954 dual treatment).

Repeated measures analysis of variance of the static weight bearing data, followed by Tukey post-hoc hypothesis tests (Table 2), demonstrated that treatment with R-954, or both BQ-123 and R-954, significantly ameliorated nociceptive tolerance in ACLT animals over the study period, as compared to saline-treated positive controls (0.0001 ≤ *P *≤ 0.0002). When administered alone, BQ-123 did not result in statistically significant increased nociceptive tolerance (*P *= 0.1847). Sham surgery was found to be slightly less nociceptive than ACL transection (*P *= 0.019), confirming that ACLT is necessary for a maximal nociceptive response. Furthermore, nociception in the sham-operated animals was comparable to unoperated animals, with no statistically significant difference calculated (*P *= 0.8746).

**Table 2:** Static weight bearing post-hoc tests

Contrast	Estimate	Standard error	*z*-score	***P*(*>***|***z***|**)**
None/Saline vs Sham/Saline	- 5.1697	1.9903	- 2.597	0.8746
Sham/Saline vs ACLT/Saline	6.5667	2.0155	3.258	0.019
ACLT/BQ-123 vs ACLT/Saline	2.5845	1.8841	1.372	0.1847
ACLT/R-954 vs ACLT/Saline	0.6951	1.9669	0.353	0.0002
ACLT/BQ-123+R-954 vs ACLT/Saline	0.2784	1.8503	0.15	0.0001

Tukey post-hoc tests were conducted following repeated measures ANOVA of the static weight bearing data. From left to right, the table columns present the contrast of interest, the parameter estimate from the linear matrix model, the standard error of that estimate, the standard *z*-score, and the associated *P*-value.

### Antagonist treatment improved radiological indices of OA

**Figure 2:**
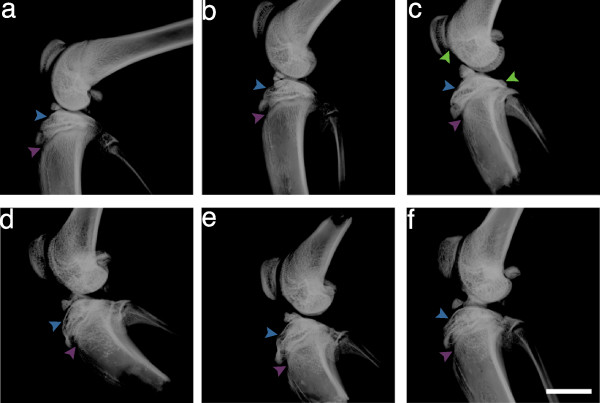
**Antagonist treatment improves radiological indices of OA: X-ray results**. **(a) **No surgery and saline treatment; **(b) **sham surgery and saline treatment; **(c) **ACLT and saline treatment; **(d) **ACLT and BQ-123 treatment; **(e) **ACLT and R-954 treatment; **(f) **ACLT and BQ-123+R-954 dual treatment. Blue arrows indicate tibial plateau, purple arrows indicate subchondral bone, and green arrows indicate osteophytes. Sagittal views. Scale bar, 1 cm.

**Figure 3:**
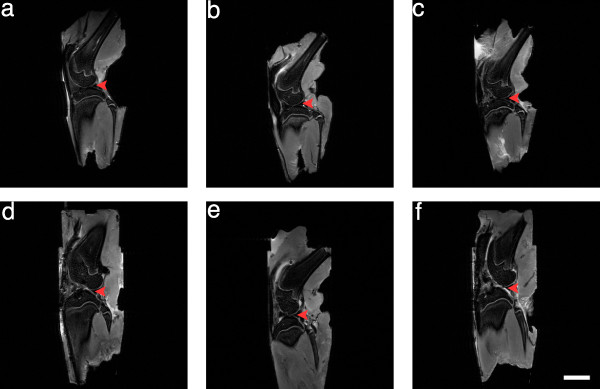
**Antagonist treatment improves radiological indices of OA: MR results**. **(a) **No surgery and saline treatment; **(b) **sham surgery and saline treatment; **(c) **ACLT and saline treatment; **(d) **ACLT and BQ-123 treatment; **(e) **ACLT and R-954 treatment; **(f) **ACLT and BQ-123+R-954 dual treatment. Red arrows indicate articular cartilage. Sagittal views. Scale bar, 1 cm.

Right knee joints were dissected at the end of the study period and imaged by DX (Figure 2) and MR (Figure 3) to examine the radiological effects of antagonist treatments. ACLT rapidly induced radiological evidence of OA: knee joints showed signs of degradation such as subchondral bone remodeling, osteophyte formation (Figure 2c and Table 3), cartilage layer thinning (Figure 3c), and lengthened cartilage T_2 _(Table 4). Neither sham surgery nor intra-articular injection affected joint radiomorphology (Figures 2a,b and 3a,b). DX analysis of antagonist-treated knee joints showed less subchondral bone remodeling and heterotopic ossification than saline-treated animals (Figure 2d,e,f and Table 3). Dual ETA/BKB1 antagonism appeared to be slightly more protective than single antagonism: less subchondral bone remodeling and greater trabecular integrity was observed in the dual-antagonist-treated animals than in the single-antagonist-treated animals. Radiological scoring of the DX views for a panel of OA joint degenerative changes (Table 3 and Additional file 5) demonstrated that treatment with BQ-123, R-954, or both, significantly ameliorated radiological indices of disease progression in ACLT animals, as compared to saline-treated positive controls (0.0020 ≤ *P *≤ 0.0214, one-way ANOVA with Holm post-hoc). MR analysis of knee joints revealed that antagonist-treated animals had greater cartilage thickness and fewer cartilage lesions (Figure 3d,e,f), as well as shorter cartilage T_2 _(Table 4, statistical significance not achieved) than saline-treated ACLT animals. These data suggest that antagonist treatment protected joint radiomorphology after ACLT.

**Table 3:** OA radiological scores

Group number	Surgery	Treatment	Mean total radiological score	SD
1	None	Saline	0.25	0.50
2	Sham	Saline	1.16	0.75
3	ACLT	Saline	4.86	1.68
4	ACLT	BQ-123	2.83	1.47^a^
5	ACLT	R-954	2.50	1.22^b^
6	ACLT	BQ-123+R-954	2.67	1.03^c^

Radiological scoring of the DX views of the knee joints [42,43] indicated that antagonist treatment protected joint radiomorphology after ACLT. One-way ANOVA with Holm post-hoc: ^a^*P *= 0.0214, ACLT/BQ-123 treatment versus ACLT/saline treatment; ^b^*P *= 0.0020, ACLT/R-954 treatment versus ACLT/saline treatment; ^c^*P *= 0.0125, ACLT/BQ-123+R-954 dual treatment versus ACLT/saline treatment.

**Table 4:** Cartilage mean T_2 _values

Group number	Surgery	Treatment	**Mean T**_**2 **_**value (ms)**
1	None	Saline	51.60
2	Sham	Saline	52.12
3	ACLT	Saline	64.38
4	ACLT	BQ-123	63.23
5	ACLT	R-954	61.13
6	ACLT	BQ-123+R-954	56.57

Cartilage mean T_2 _values in milliseconds were calculated for all conditions using OsiriX software (version 3.7.1) [41]. Statistical significance in between groups was not achieved.

### Antagonism protects joint histomorphology

**Figure 4:**
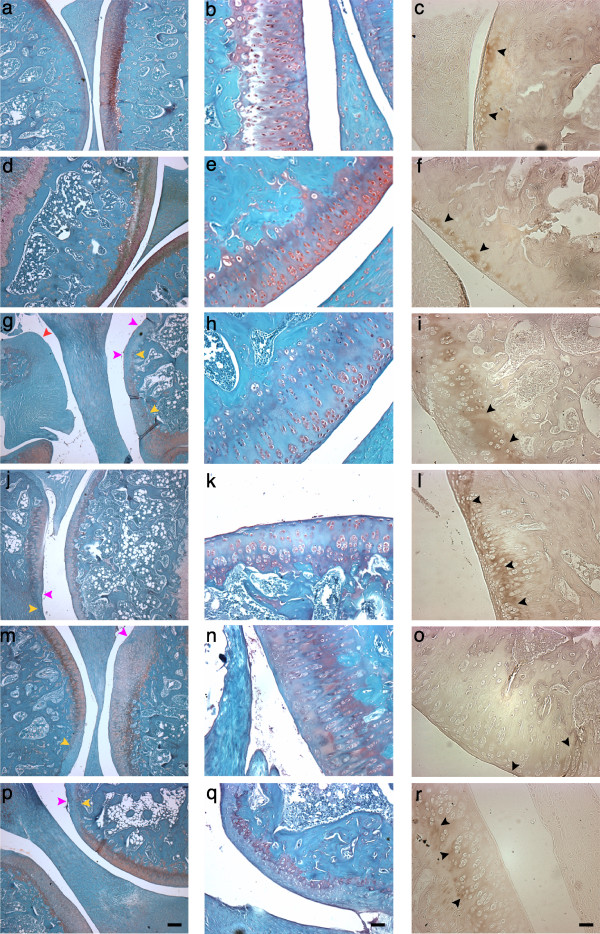
**Antagonist treatment protects joint histomorphometry**. Sagittal sections. Left and middle columns, Safranin O/Fast Green FCF staining. Right column, type II collagen immunohistochemistry. Left column, low-power magnification: scale bar, 200 *μ*m; original magnification 50 ×. Middle and right columns, high-power magnification: scale bar, 50 *μ *m; original magnification 200 ×. Conditions by rows: **(a)**, **(b)**, **(c) **no surgery and saline treatment; **(d)**, **(e)**, **(f) **sham surgery and saline treatment; **(g)**, **(h)**, **(i) **ACLT and saline treatment; **(j)**, **(k)**, **(l) **ACLT and BQ-123 treatment; **(m)**, **(n)**, **(o) **ACLT and R-954 treatment; **(p)**, **(q)**, **(r) **ACLT and BQ-123+R-954 dual treatment. Yellow arrows indicate loss of Safranin O staining, purple arrows indicate cartilage notching, and red arrow indicates an osteophyte. Black arrows indicate type II collagen immunostaining.

To investigate the effects of ETA and/or BKB1 antagonist treatment on histological indices of disease, rat knee joints were processed to assess cartilage proteoglycan content and joint histomorphology (Figure 4 left and middle columns). ACLT saline-treated animals lost most proteoglycan staining when examined at eight weeks post-operatively, with severe articular surface disruptions and osteophyte formation (Figure 4g,h). In contrast, cartilage proteoglycans were detected in the knees of ETA and/or BKB1 antagonist-treated animals (Figures 4j,k,m,n and 4p,q), indicating that treatment protects cartilage structural components. As well, articular surface integrity was preserved to a greater extent, with dual antagonism appearing to be most protective (Figure 4p,q). Neither sham surgery nor intra-articular injection of saline vehicle negatively affected joint histomorphology (Figures 4a,b and 4d,e). Mean OARSI scores (Table 5 and Additional file 6) indicate that ETA and/or BKB1 antagonist treatment significantly reduced the amount of affected joint tissue and the degree of histopathology, as compared to saline-treated positive controls (*P *< 0.0001 for all comparisons, one-way ANOVA with Holm post-hoc).

**Table 5:** OARSI histopathology scores

Group number	Surgery	Treatment	Mean OARSI score	SD
1	None	Saline	0.43	0.53
2	Sham	Saline	0.50	1.00
3	ACLT	Saline	17.00	5.77
4	ACLT	BQ-123	4.75	0.96^a^
5	ACLT	R-954	4.25	2.02^b^
6	ACLT	BQ-123+R-954	3.50	2.89^c^

Four slides per condition were scored by two blinded examiners using the OARSI histopathology assessment system [46]. Results were averaged and are presented as mean scores per condition. Inter-examiner variation was within ± 5%. One-way ANOVA with Holm post-hoc: ^a^*P *= 0.000017, ACLT/BQ-123 treatment versus ACLT/saline treatment; ^b^*P *= 0.00001, ACLT/R-954 treatment versus ACLT/saline treatment; ^c^*P *= 0.0000048, ACLT/BQ-123+R-954 dual treatment versus ACLT/saline treatment.

Type II collagen, the major structural collagen of cartilage, was detected by immunohistochemistry (Figure 4 right column). ACLT saline-treated animals displayed significant losses of articular surface type II collagen (Figure 4i) with some localization in the deep zones of cartilage, reflecting cartilage remodeling processes. Animals treated with ETA and/or BKB1 antagonists (Figure 4l,o,r) displayed varying degrees of protection, retaining some type II collagen staining. Neither sham surgery nor intra-articular injection of saline vehicle negatively affected joint type II collagen expression (Figures 4c and 4f): protein was localized in the superficial zone of articular cartilage, indicating functional joint tissue.

## Discussion

In the present study, we investigated whether antagonism of ETA and/or BKB1 could slow and/or prevent osteoarthritic cartilage degradation and joint nociception in a rat surgical model of OA. We provide several lines of evidence that suggest protective effects of ETA and/or BKB1 antagonism *in vivo*: BKB1 antagonist treatment improved hind limb nociceptive tolerance, and both ETA and/or BKB1 antagonist treatment ameliorated radiological indices of disease, and protected articular cartilage and bone histomorphometry.

The most interesting finding of our study is that nociceptive tolerance was augmented in our model after BKB1 antagonist treatment, with faster post-operative recovery than vehicle-treated controls. These results are consistent with other reports [19], where local treatment with BKB1 receptor antagonists reduced overt acute joint nociception. We extend this finding to the dual antagonist treatment approach to male animals in a model of chronic pain, as well as relating it to measures of joint integrity by radiology and histology. Low-grade joint pain is the most common reason for patient presentation, and is often the major debilitating factor in OA cases [47,48]. Thus, the anti-nociceptive effects of BKB1 antagonism make this treatment strategy attractive. Surprisingly, single ETA antagonism was relatively ineffective at diminishing joint nociception in our model. This finding, which contradicts our initial hypothesis, suggests that ETA potentiation of ET-1-induced joint pain [14] may not be direct, especially in a chronic inflammatory state.

We found that single and dual ETA/BKB1 antagonist treatments decreased radiological disease indices, in terms of osteophyte formation, cartilage thinning, and subchondral bone remodeling, with dual antagonism being most protective. As well, cartilage T_2_, increased in ACLT animals, was decreased by antagonist treatment, which indicates a cartilage-preserving effect. Longer cartilage transverse relaxation times are an indicator of cartilage degradation; this MR parameter is indicative of cartilage composition and integrity [49-51]. Radiographic evidence is the main criterion for OA diagnosis and progression [52,53]. The most common clinical diagnostic test is via X-ray of the affected joint: joint space narrowing as measured on X-ray is often used as a longitudinal marker of disease evolution. It is difficult to directly compare radiological parameters between human and rat knees due to the quadrupedal nature of the animal and the markedly different radiological anatomy that this entails [54]. However, we were able to detect radiological evidence of OA progression in ACLT animals, as has been described in similar studies [44,55].

OA induction in rat knees leads to a rapid decrease in cartilage proteoglycan staining, along with articular surface disruption and osteophyte formation [26,27]. ETA/BKB1 antagonist treatment protected the proteoglycan content of the joint and preserved articular surface integrity. Furthermore, there was some protection of type II collagen protein expression. This allowed the joint cartilage to retain its normal biophysical properties, as cartilage proteoglycans are responsible, along with collagen, for retaining water in the tissue, which provides spring and resilience [56,57]. These findings likely suggest that the protection of cartilage proteoglycans, collagens, and articular surface histomorphology may be one explanation for the increased pain tolerance observed in antagonist-treated animals; our results concur with those of other reports, which correlated the preservation of articular cartilage proteoglycan staining with pain tolerance behavior [26].

The ET-1 and BK systems are involved in joint tissue inflammation and nociception, concomitant with pro-inflammatory mediators. However, exploration of potential therapeutic targets in these systems has been modest: the main classes of disease-modifying osteoarthritis drugs currently in development include cytokine and matrix metalloproteinase inhibitors, anti-resorptives, and growth factors [58]. To our knowledge, the only clinical trial of a drug targeting a vasoactive factor in OA is the bradykinin receptor B2 antagonist Icatibant, by Sanofi-Aventis [59]. This drug is no longer in clinical development [60], due to mixed results: while it provided local analgesia in knee OA, no anti-inflammatory effect could be detected [61]. Our results suggest that ETA and BKB1 represent novel therapeutic targets in OA. Specific receptor antagonists could be tested in clinical trials for OA pain and tissue damage.

## Conclusions

Using a rat surgically induced model of OA, we demonstrated that local treatment with specific peptide antagonists of ETA and/or BKB1 may slow or stabilize the development of radiomorphological and histomorphological changes occurring in OA pathogenesis. Furthermore, we showed that BKB1 antagonist treatment accelerated recovery of, and improved longitudinally, nociceptive tolerance in ACLT animals. Taken together, our results indicate that blocking ETA and BKB1 improves OA prognostic indices, which implies that defective signaling might play a role in chronic OA pain. Our results also raise the possibility of targeted receptor antagonism as a relevant therapeutic option. Further studies are required to understand the mechanisms underlying the exact nature of receptor cross-regulation and synergism.

## Abbreviations

ACLT: anterior cruciate ligament transection; ANOVA: analysis of variance; BK: bradykinin; BKB1: bradykinin receptor B1; BKB2: bradykinin receptor B2; DX: digital micro-X-ray; ET-1: endothelin-1; ETA: endothelin receptor type A; ETL: echo train length; MR: magnetic resonance; MSME: multislice multiecho; OA: osteoarthritis; OARSI: Osteoarthritis Research Society International; PBS: phosphate-buffered saline; RARE: rapid acquisition with relaxation enhancement; RF: radio frequency; SD, standard deviation; TE: echo time; TR: repetition time.

## Competing interests

Intellectual property rights (GNK, PS, FM) of the dual-antagonist treatment strategy are protected through Univalor, the technology transfer corporation of Université de Montréal. PS holds patents relating to the preparation and use of R-954. CZ and BV declare that they have no competing interests.

## Authors' contributions

GNK designed the *in vivo *study, performed the surgeries, injections, static weight bearing measurements, dissections, and radiological analyses, analyzed the data, and wrote the paper. CZ assisted with surgeries and dissections, performed histological studies, and revised the paper. BV reverse-engineered the static weight bearing apparatus and assisted with data analysis. PS contributed the BKB1 antagonist R-954. FM conceived the study and supervised the research group. GNK gabriel.kaufman@umontreal.ca takes responsibility for the integrity of the work as a whole. All authors read and approved the final manuscript.

## Supplementary Material

Additional file 1 **Rat anterior cruciate ligament transection and intra-articular injection**. Detailed descriptions and macro photographs of rat anterior cruciate ligament transection and intra-articular injection. PDF file named Rat ACLT and IA injection.pdf (3 pages). Click here for file

Additional file 2 **Chemical structures of BQ-123 and R-954**. 2D chemical structures of selective ETA peptide antagonist BQ-123 (left) and selective BKB1 peptide antagonist R-954 (right). PDF file named antagonist structures.pdf (1 page). Click here for file

Additional file 3 **Design diagrams for static weight bearing apparatus**. Original design diagrams for static weight bearing apparatus. Labels in French. Auto-drafted using CATIA V5 R19. PDF file named static weight bearing apparatus design diagrams.pdf (4 pages). Click here for file

Additional file 4 **Static weight bearing apparatus in use**. Static weight bearing apparatus with rat positioned for measurements. A, side view; B, angle view; C, front view. PDF file named static weight bearing apparatus photos.pdf (1 page). Click here for file

Additional file 5 **OA radiological scores**. Unblinded raw data for the OA radiological scores, presented as averaged scores for each parameter. CSV file named radiological scores.csv. Click here for file

Additional file 6 **OARSI histopathology scores**. Unblinded raw data for the OARSI histopathology scores, presented as averaged scores for each parameter. CSV file named OARSI scores.csv. Click here for file
