# Supplementary material for: Nociceptive tolerance is improved by bradykinin receptor B1 antagonism and joint morphology is protected by both endothelin type A and bradykinin receptor B1 antagonism in a surgical model of osteoarthritis
Source: Arthritis Res Ther. 2011 May 16;13(3):R76. doi: 10.1186/ar3338 (PMC3218886; doi:10.1186/ar3338)
Supplement: Additional file 1 — Rat anterior cruciate ligament transection and intra-articular injection. Detailed descriptions and macro photographs of rat anterior cruciate ligament transection and intra-articular injection. PDF file named Rat ACLT and IA injection.pdf (3 pages). [file ar3338-S1.PDF]

Additional File 1
Rat anterior cruciate ligament transection and intra-articular injection

Surgical technique

OA was induced by surgical transection of the right anterior cruciate ligament. Animals were anaesthetized with inhaled isoflurane (3% / L O_2 induction in chamber, 2% / L O_2 maintenance with face-mask), and prepared for surgery by clipping the hair over the ventral and medial aspects of the right leg from hindpaw to hip. The skin was disinfected with povidone-iodine, and a 3-cm incision was made medial to the patellar tendon (Figure AF1.1A). The subcutaneous tissue and muscle were then incised and the patella laterally sublaxed; the joint capsule was opened with the limb hyperextended (Figure AF1.1B). With the limb in full flexion, the anterior cruciate ligament was visualized by blunt dissection, and sectioned by a latero-medial cut parallel to the tibial plateau, using a #11 scalpel blade (Figure AF1.1C-D). Transection was confirmed with the anterior drawer test (Figure AF1.1E-F: E depicts the knee before, and F shows an anterior drawer). The patella was then replaced, and the limb extended (Figure AF1.1G). The joint capsule (Figure AF1.1H) and muscle layers (Figure AF1.1I) were closed with 4-0 polygalactin absorbable suture (horizontal mattress stitch, Figure AF1.1J). 50 μL of lidocaine was then injected into the joint capsule to provide local analgesia. Skin was closed with steel surgical staples (Figure AF1.1K). Post-operative hydration (6 mL/kg saline) and systemic analgesia (0.1 mg/kg buprenorphine HCl) were provided by subcutaneous injection. Surgical staples were removed 14 days post-operatively (Figure AF1.1L). Sham surgery consisted of all of the above except ligament transection.

Intra-articular injection

Over the course of two months post-operatively, animals were treated by weekly intra-articular injections of ETA and/or BKB1 specific peptide antagonists, both, or saline vehicle. Drugs were injected into the right knee at a dose of 30 nmol in a volume of 50 μL. Injections were performed under isoflurane anaesthesia, using a 28G needle (Figure AF1.2).

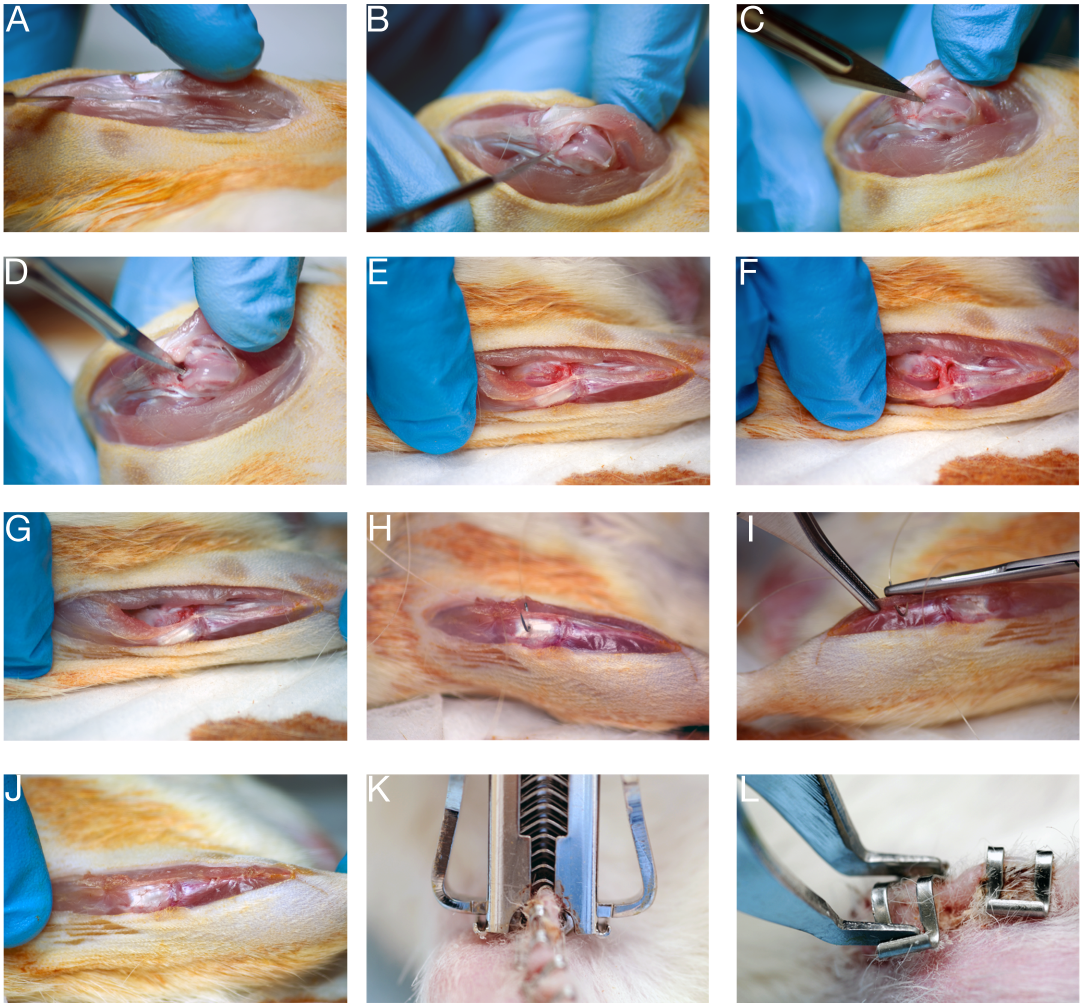

Figure 1: Rat ACLT surgical steps. Photos were taken with a 300-mm macro lens (approximate magnification 2×).

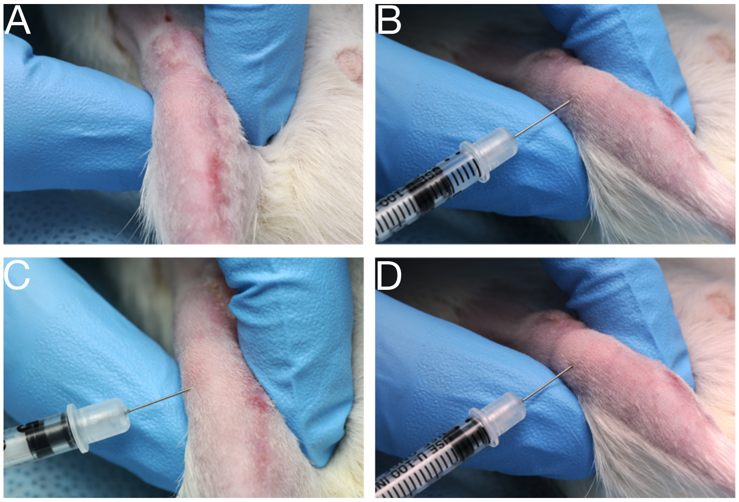

Figure 2: Intra-articular injection into the rat knee. A, the shaved knee is maintained in extension. B, the needle is inserted under the patellar tendon into the joint space. C, the syringe plunger is depressed slowly. D, successful injection is detected by a momentary swelling of the articular space. Photos were taken with a 300-mm macro lens (approximate magnification 2×).
