# Supplementary figures and images for: Nociceptive tolerance is improved by bradykinin receptor B1 antagonism and joint morphology is protected by both endothelin type A and bradykinin receptor B1 antagonism in a surgical model of osteoarthritis
Source: Arthritis Res Ther. 2011 May 16;13(3):R76. doi: 10.1186/ar3338 (PMC3218886; doi:10.1186/ar3338)

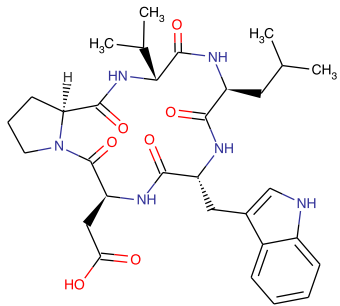

BQ-123

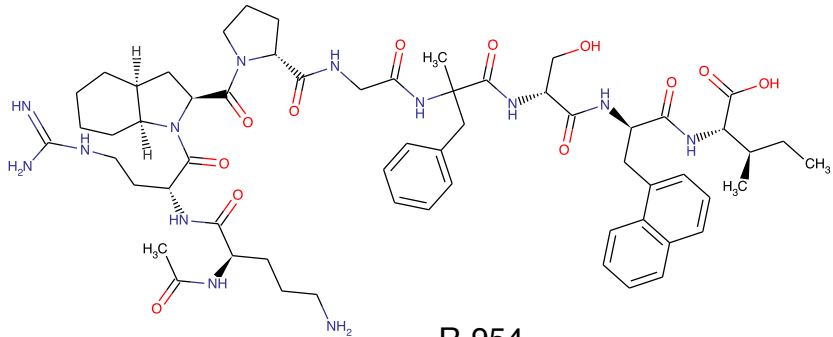

R-954

Supplement: Additional file 2 — Chemical structures of BQ-123 and R-954. 2D chemical structures of selective ETA peptide antagonist BQ-123 (left) and selective BKB1 peptide antagonist R-954 (right). PDF file named antagonist structures.pdf (1 page). [file ar3338-S2.PDF]

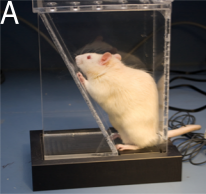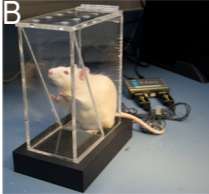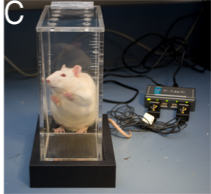

Supplement: Additional file 4 — Static weight bearing apparatus in use. Static weight bearing apparatus with rat positioned for measurements. A, side view; B, angle view; C, front view. PDF file named static weight bearing apparatus photos.pdf (1 page). [file ar3338-S4.PDF]
