# Supplementary material for: Nociceptive tolerance is improved by bradykinin receptor B1 antagonism and joint morphology is protected by both endothelin type A and bradykinin receptor B1 antagonism in a surgical model of osteoarthritis
Source: Arthritis Res Ther. 2011 May 16;13(3):R76. doi: 10.1186/ar3338 (PMC3218886; doi:10.1186/ar3338)
Supplement: Additional file 3 — Design diagrams for static weight bearing apparatus. Original design diagrams for static weight bearing apparatus. Labels in French. Auto-drafted using CATIA V5 R19. PDF file named static weight bearing apparatus design diagrams.pdf (4 pages). [file ar3338-S3.PDF]

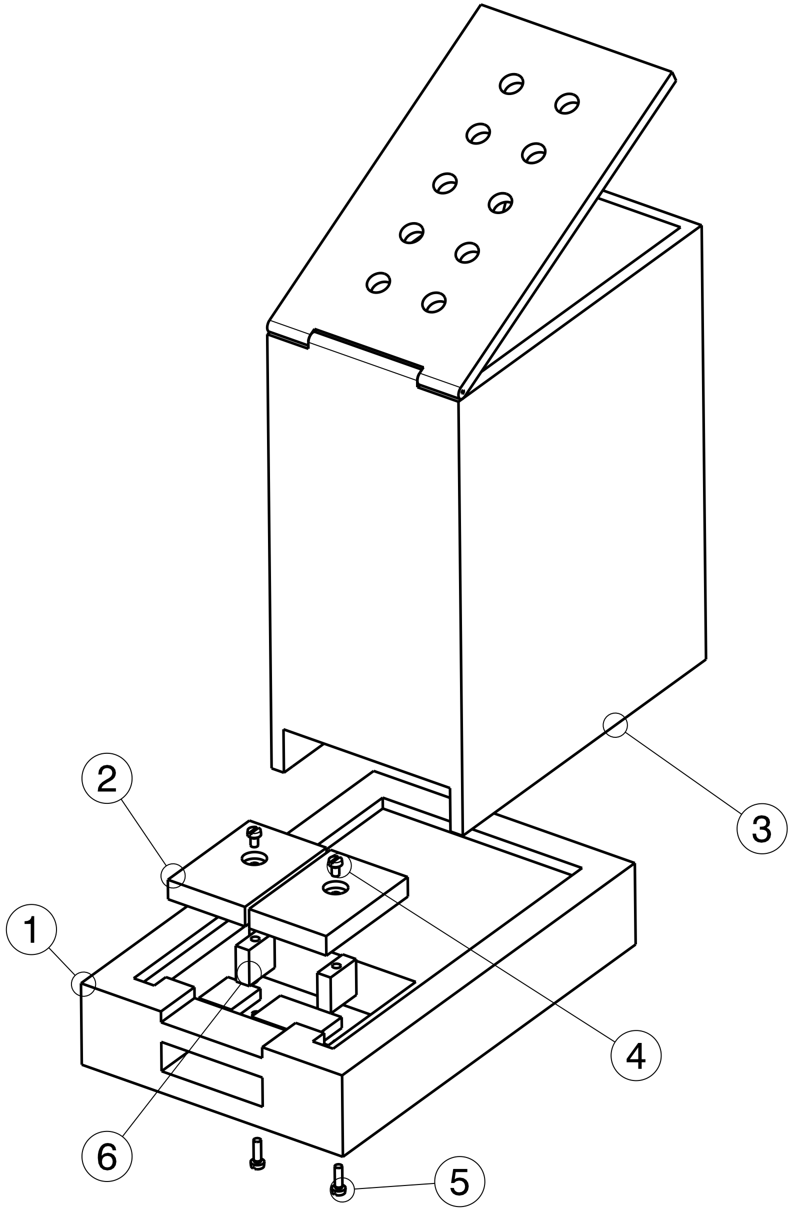

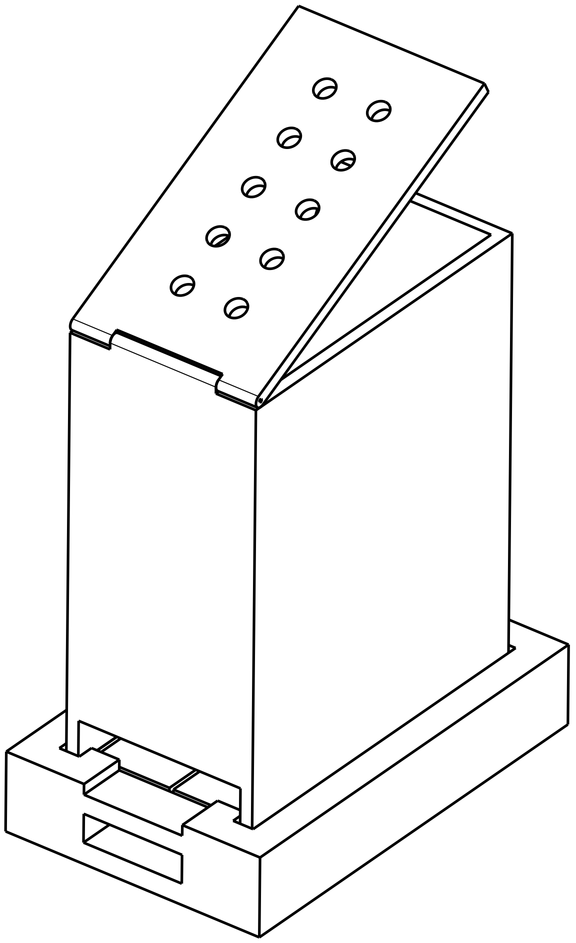

No.	No. Dessin	Nom du composant	Quantité
6	NON USINÉ	Cellules de charge (FUTEK MODEL LSB200 - 250g)	2
5	STANDARD	Spaenaur 382-079 : M3 x 10 mm	2
4	STANDARD	Spaenaur 382-078 : M3 x 6 mm	2
3	LBMO-003	Boîte de contention	1
2	LBMO-002	Plaque support	2
1	LBMO-001	Plateforme support	1

Centre de recherche (STE JUSTINE)

LABORATOIRE DE BIOLOGIE MOLÉCULAIRE ORTHOPÉDIQUE

NOTE<br>---	TITRE<br>Assemblage - Appareil d'incapacitance statique		
	No. DESSIN<br>LBMO-000	MATÉRIAU<br>---	DIMENSIONS<br>---
CONTACT<br>Gabriel KAUFMAN<br>(514) 345 4691 # 5757<br>(gabriel.kaufman@umontreal.ca)	DESSINATEUR<br>Barthélémy VALTEAU	DATE<br>23/07/09	
	APPROUVÉ<br>F. MOLDOVAN	ÉCHELLE<br>---	

No. Révision	Description	Révisé	Approuvé	Date
...	...	...	...	...
...	...	...	...	...

RÉVISIONS

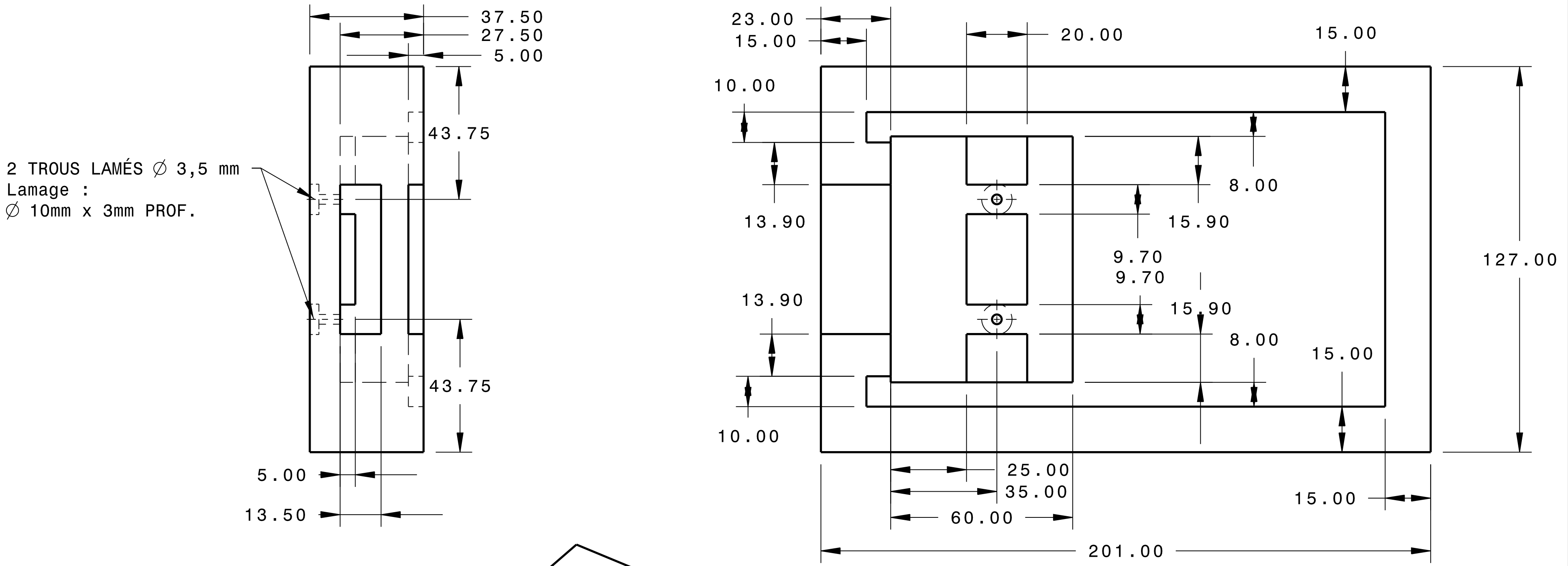

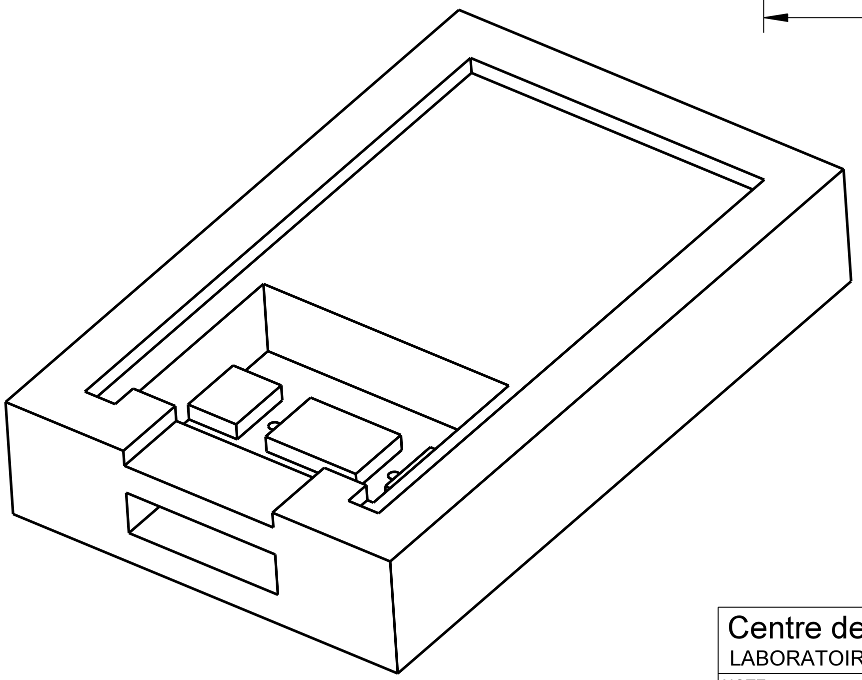

...	...	...	...	...
...	...	...	...	...
No. Révision	Description	Révisé	Approuvé	Date

RÉVISIONS

Centre de recherche (STE JUSTINE)

LABORATOIRE DE BIOLOGIE MOLÉCULAIRE ORTHOPÉDIQUE

NOTE<br>...	TITRE<br>Plateforme support		
	No. DESSIN<br>LBMO-001	MATÉRIAU<br>UHMWPE Noir	DIMENSIONS<br>mm
CONTACT<br>Gabriel KAUFMAN<br>(514) 345 4691 # 5757<br>(gabriel.kaufman@umontreal.ca)	DESSINATEUR<br>Barthélémy VALTEAU	DATE<br>23/07/09	TOLÉRANCES GÉNÉRALES<br>X.XX ± 0.07mm<br>Angle ± 0.5°
	APPROUVÉ<br>F. MOLDOVAN	ÉCHELLE<br>1:2	

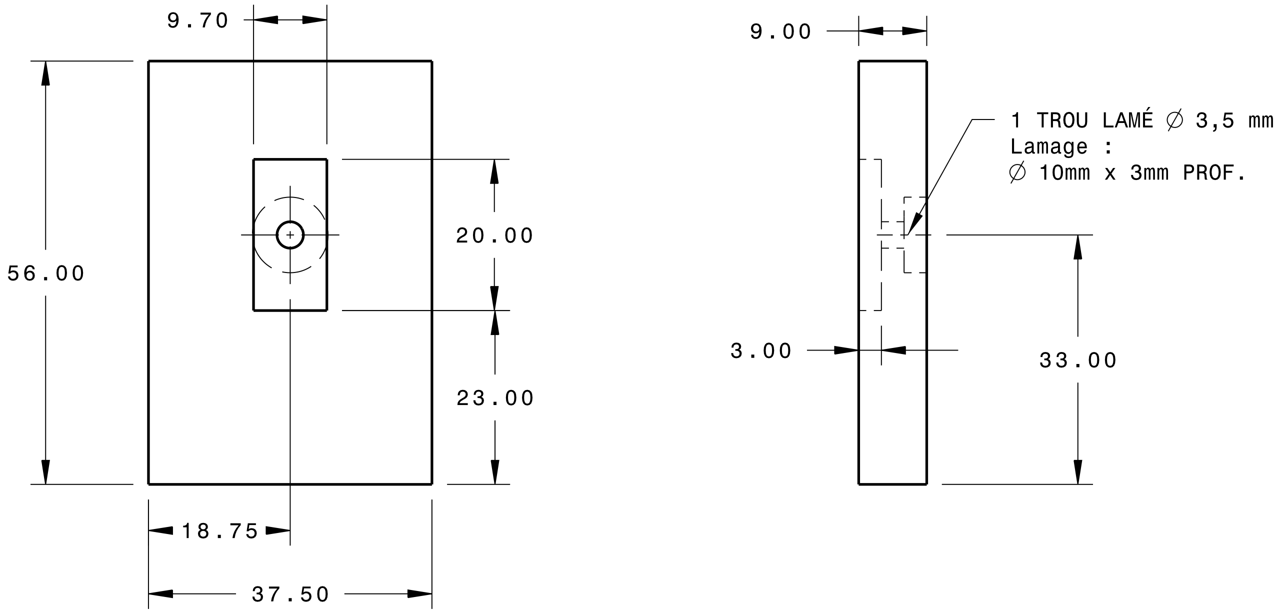

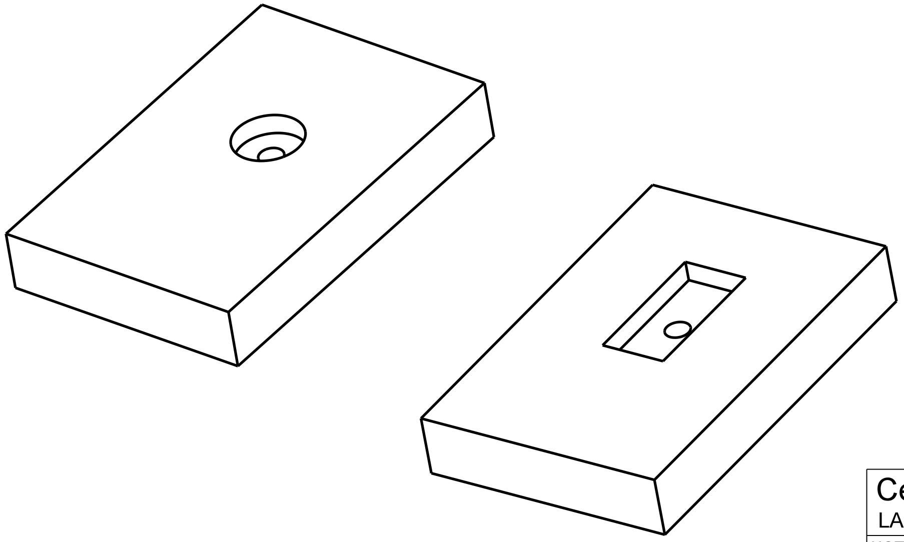

No. Révision	Description	Révisé	Approuvé	Date
...	...	...	...	...
...	...	...	...	...

RÉVISIONS

Centre de recherche (STE JUSTINE)

LABORATOIRE DE BIOLOGIE MOLÉCULAIRE ORTHOPÉDIQUE

NOTE<br>...	TITRE<br>Plaque support		
	No. DESSIN<br>LBMO-002	MATÉRIAU<br>UHMWPE Noir	DIMENSIONS<br>mm
CONTACT<br>Gabriel KAUFMAN<br>(514) 345 4691 # 5757<br>(gabriel.kaufman@umontreal.ca)	DESSINATEUR<br>Barthélémy VALTEAU	DATE<br>23/07/09	TOLÉRANCES GÉNÉRALES<br>X.XX ± 0.07mm<br>Angle ± 0.5°
	APPROUVÉ<br>F. MOLDOVAN	ÉCHELLE<br>1:1	

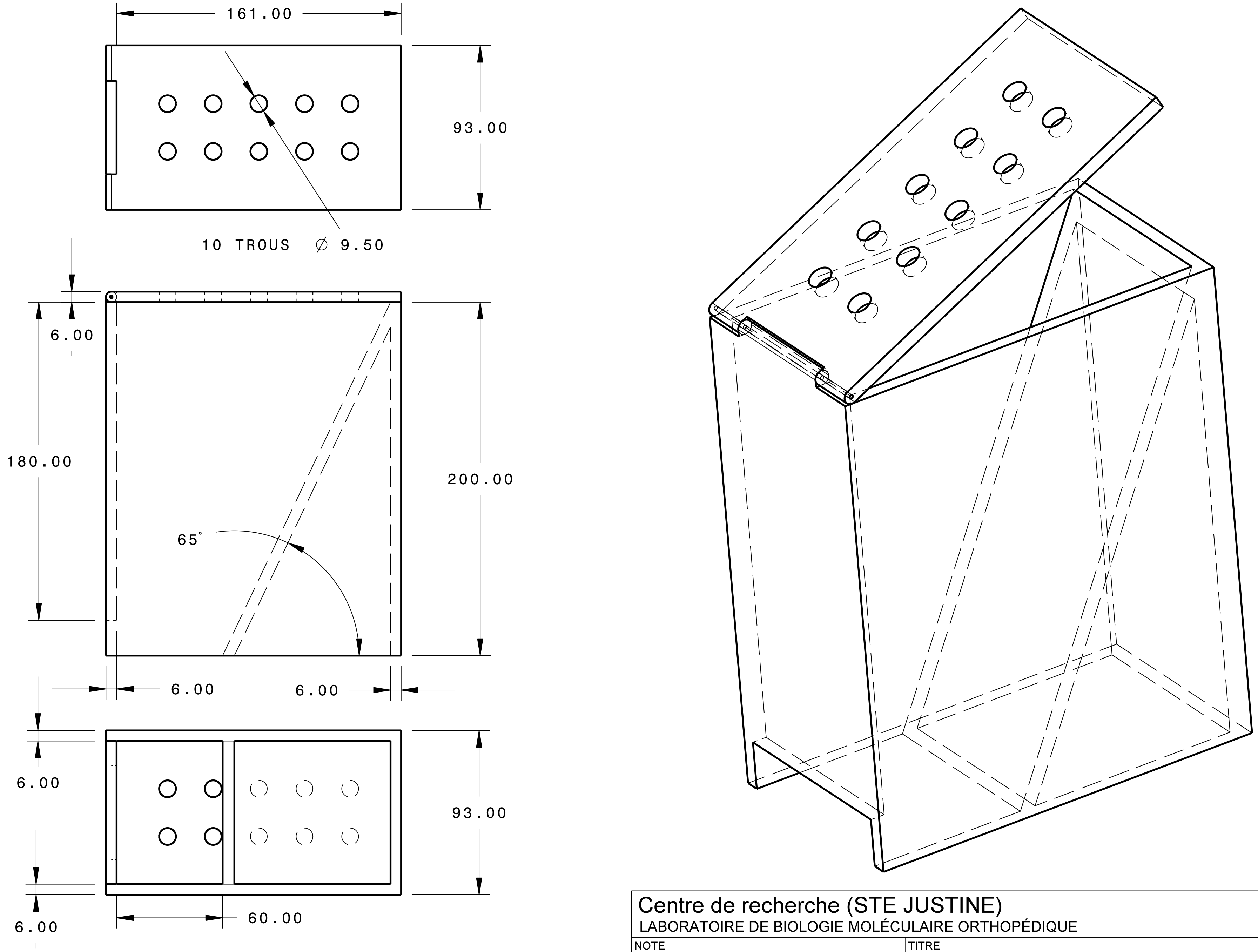

Centre de recherche (STE JUSTINE)

LABORATOIRE DE BIOLOGIE MOLÉCULAIRE ORTHOPÉDIQUE

NOTE<br>---	TITRE<br>Boîte de contention		
	No. DESSIN<br>LBMO-003	MATÉRIAU<br>Plexiglass	DIMENSIONS<br>mm
CONTACT<br>Gabriel KAUFMAN<br>(514) 345 4691 # 5757<br>(gabriel.kaufman@umontreal.ca)	DESSINATEUR<br>Barthélémy VALTEAU	DATE<br>23/07/09	TOLÉRANCES GÉNÉRALES<br>X.XX ± 1mm<br>Angle ± 0.5°
	APPROUVÉ<br>F. MOLDOVAN	ÉCHELLE<br>1:3	

No. Révision	Description	Révisé	Approuvé	Date
...	...	...	...	...
...	...	...	...	...

RÉVISIONS
